# Comparing artificial neural network training algorithms to predict length of stay in hospitalized patients with COVID-19

**DOI:** 10.1186/s12879-022-07921-2

**Published:** 2022-12-09

**Authors:** Azam Orooji, Mostafa Shanbehzadeh, Esmat Mirbagheri, Hadi Kazemi-Arpanahi

**Affiliations:** 1 Department of Medical Informatics, Department of Advanced Technologies, School of Medicine, North Khorasan University of Medical Science (NKUMS), North Khorasan, Iran; 2 Department of Health Information Management, Department of Health Information Technology, School of Paramedical, Ilam University of Medical Sciences, Ilam, Iran; 3 Department of Health Information Management, Iran University of Medical Sciences, Tehran, Iran; 4 Department of Health Information Management, Department of Health Information Technology, Abadan University of Medical Sciences, Abadan, Iran; 5 Department of Health Information Management, Student Research Committee, Abadan University of Medical Sciences, Abadan, Iran

**Keywords:** COVID-19, Coronavirus, Artificial neural networks, Training algorithms, Length of stay

## Abstract

**Background:**

The exponential spread of coronavirus disease 2019 (COVID-19) causes unexpected economic burdens to worldwide health systems with severe shortages in hospital resources (beds, staff, equipment). Managing patients’ length of stay (LOS) to optimize clinical care and utilization of hospital resources is very challenging. Projecting the future demand requires reliable prediction of patients’ LOS, which can be beneficial for taking appropriate actions. Therefore, the purpose of this research is to develop and validate models using a multilayer perceptron-artificial neural network (MLP-ANN) algorithm based on the best training algorithm for predicting COVID-19 patients' hospital LOS.

**Methods:**

Using a single-center registry, the records of 1225 laboratory-confirmed COVID-19 hospitalized cases from February 9, 2020 to December 20, 2020 were analyzed. In this study, first, the correlation coefficient technique was developed to determine the most significant variables as the input of the ANN models. Only variables with a correlation coefficient at a P-value < 0.2 were used in model construction. Then, the prediction models were developed based on 12 training algorithms according to full and selected feature datasets (90% of the training, with 10% used for model validation). Afterward, the root mean square error (RMSE) was used to assess the models’ performance in order to select the best ANN training algorithm. Finally, a total of 343 patients were used for the external validation of the models.

**Results:**

After implementing feature selection, a total of 20 variables were determined as the contributing factors to COVID-19 patients’ LOS in order to build the models. The conducted experiments indicated that the best performance belongs to a neural network with 20 and 10 neurons in the hidden layer of the Bayesian regularization (BR) training algorithm for whole and selected features with an RMSE of 1.6213 and 2.2332, respectively.

**Conclusions:**

MLP-ANN-based models can reliably predict LOS in hospitalized patients with COVID-19 using readily available data at the time of admission. In this regard, the models developed in our study can help health systems to optimally allocate limited hospital resources and make informed evidence-based decisions.

## Background

Coronavirus disease 2019 (COVID-19) is a very contagious viral infection that has so far continued to spread rapidly around the world and has become a serious global health problem. The rapid outbreak of COVID-19 exposed healthcare organizations to hospital resource shortages and the exhaustion of frontline healthcare workers [1–6]. So far, clinical manifestations have shown substantial heterogeneity among different patients, ranging from asymptomatic or mild flu-like symptoms to severe respiratory illness and pneumonia, intensive care unit (ICU) hospitalization, multi-organ failure (MOF), and ultimately death [7]. The high transmission rates of COVID-19, the emergence of new variants, and unknown clinical patterns put immense pressure on health systems. As a result, there is a drastic increase in the number of patients seeking medical attention and a surge in hospitalizations [8, 9]. This overcrowding raises serious concerns regarding the potential impact of the spread of the virus, especially on health systems with severe resource constraints in low-and middle-income countries (LMICs) [10, 11]. During this pandemic crisis, to make healthcare more affordable and prevent the overwhelming of hospitals, it is crucial to adopt objective and evidence-based interventions for the effective use of medical facilities available in hospitals (e.g., hospital beds and respiratory ventilators, among others) [12].

As the pandemic worsens, identifying the consequent needs of patients and service providers has become essential. It is necessary to anticipate how long each case will need inpatient services [13, 14]. Length of stay (LOS) is an important measure of health services quality and resource utilization, which is often used to decrease health care charges, especially given the increase in health care costs [15, 16]. From clinicians’ perspective, predicting LOS has become significantly critical during the COVID-19 epidemic for reducing the risk of adverse events, such as poor nutritional levels, community spread, adverse drug events, and other clinical problems. Furthermore, from the hospital management point of view, LOS is one of the basic measures to assess the performance of healthcare quality services, care planning, hospital staffing, resource allocation, aid in triaging, and appointment scheduling [17–20]. Accurate prediction of long LOS of patients hospitalized with COVID-19 as well as the determination of the influencing factors can contribute to optimal management and utilization of limited hospital resources. In addition, by predicting the LOS metrics, policymakers and clinicians can redesign their clinical pathways and recognize the bottlenecks for maximizing the use of medical resources [21–23]. However, LOS may be affected by many factors and its prediction can be challenging, especially in complex, novel, and ambiguous medical conditions such as the current COVID-19 crisis [21, 22]. While traditional statistical methods have been employed to forecast hospital LOS, their efficacy is restricted by the high-dimensional, censored, and heterogeneous nature of clinical data [24, 25]. Therefore, in this situation, overwhelmed health systems attempt to improve resource utilization and eliminate bottlenecks of patient hospitalization by adopting data-driven machine learning (ML) solutions [26, 27].

ML is the subarea of artificial intelligence (AI), which can be applied to analysis and inference in a large volume of retrospective datasets in order to extract distinctive relationships or identify unfamiliar patterns with minimal human intervention or any programming design [28, 29]. Furthermore, ML techniques can be employed in medical practice to increase prognostic modeling and reveal new contributing factors related to a specific target outcome to predict future or obscure trends [28, 30]. The ML technique selected in this study is the artificial neural network (ANN), which imitates the tasks of biological human neurons based on a collection of connected nodes (input-hidden-output) called artificial neurons [31, 32]. ANN can be trained to recognize and categorize complex patterns of diseases and related healthcare events through an iterative learning process. To configure the ANN, it must be trained using training patterns by changing their weights through some training algorithms. The training of ANNs can be performed by several proposed algorithms [4, 8]. Various training algorithms have been evaluated in many fields and their advantages and drawbacks have been investigated [33–36].

So far, most efforts have been focused on the application of ANN for hospital LOS prediction and determining its affecting factors [21, 37–41]. Neto et al. attempted to predict the LOS for stroke patients and reported that the ANN gained the best results with an RMSE and a mean absolute error (MAE) of 5.9451 and 4.6354, respectively [41]. Launay et al. compared two feed-forward ANNs, including multilayer perceptron (MLP) and modified MLP, for predicting prolonged LOS, and modified MLP was reported to have the best performance with a sensitivity of 62.7%, specificity of 96.6%, and an area under the receiver operating characteristic curve (AUROC) of 90.5% [42]. Morton et al. concluded that the most successful results are obtained by using the ANN technique with an RMSE of 5.9451 and MAE of 4.6354 to predict the LOS of hospitalized diabetic patients [40]. Kulkarin et al. designed an MLP-based model for predicting prolonged LOS of coronary patients with an accuracy of 90.87% [39]. Similarly, another work performed by Bacchi et al. showed that the MLP achieved the highest accuracy in the prediction of LOS with an MAE of 0.246, RMSE of 0.369, and AUC of 0.864 [43]. Kabir and Hijjry in their separate studies developed a prediction model to anticipate LOS and the results revealed that the backpropagation neural network with accuracies of 92.58% and 78.29%, respectively, outperformed all the other models in these studies [37, 38]. East et al. reported that the model developed with ANN yielded the best performance in predicting long LOS (AUC of 0.9760%) [44]. However, no studies have been performed on COVID-19 to determine the most effective ANN training algorithm and structure. This study aimed to retrospectively develop and validate ANN-based models for predicting LOS in hospitalized patients with COVID-19 according to routine clinical data available at admission time. To this end, we established and tested 12 ANN training algorithms to select the best algorithm for constructing a prediction model.

## Methods

### Study design and setting

This is a retrospective, single-center, and cross-sectional study, which was performed in 2021 for predicting LOS in hospitalized patients with COVID-19 by comparing ANN training algorithms. In this study, a COVID-19 hospital-based registry database from Ayatollah Taleghani Hospital was retrospectively analyzed to develop the ANN-based models. Ayatollah Taleghani is a large academic hospital located in Iran, Abadan City, which treats a diverse patient population.

### Study population

The analysis dataset only includes patients with a positive real-time reverse-transcriptase PCR (RT-PCR) test of throat swabs for severe acute respiratory syndrome coronavirus 2 (SARS-CoV-2) and hospital admission dates between January 9, 2020 and January 20, 2021. During this period, a total of 12,885 cases suspected of COVID-19 have been referred to Ayatollah Taleghani Hospital ambulatory and emergency departments (EDs). Of those, 3350 cases were confirmed to have COVID-19 via PCR testing. Patients discharged from the ED were excluded because their outcomes were unknown. For patients with multiple hospitalizations related to COVID-19 within the study period, only the first visit was included. Patients under the age of 18 were also excluded (n = 36). These patients should be included in the scope of pediatric exploration. Moreover, patients who died within three days of admission to the hospital were excluded from the analysis (n = 128). Since public health officials such as Centers for Disease Control and Prevention (CDC), European Centre for Disease Prevention and Control (ECDD), and the National Centre for Infectious Disease (NCID) state that three days of symptom resolution, specifically fever and respiratory symptoms, is the cutoff for safe discharge, the LOS cutoff of three days was considered [45].

### Data preparation

**Fig. 1:**
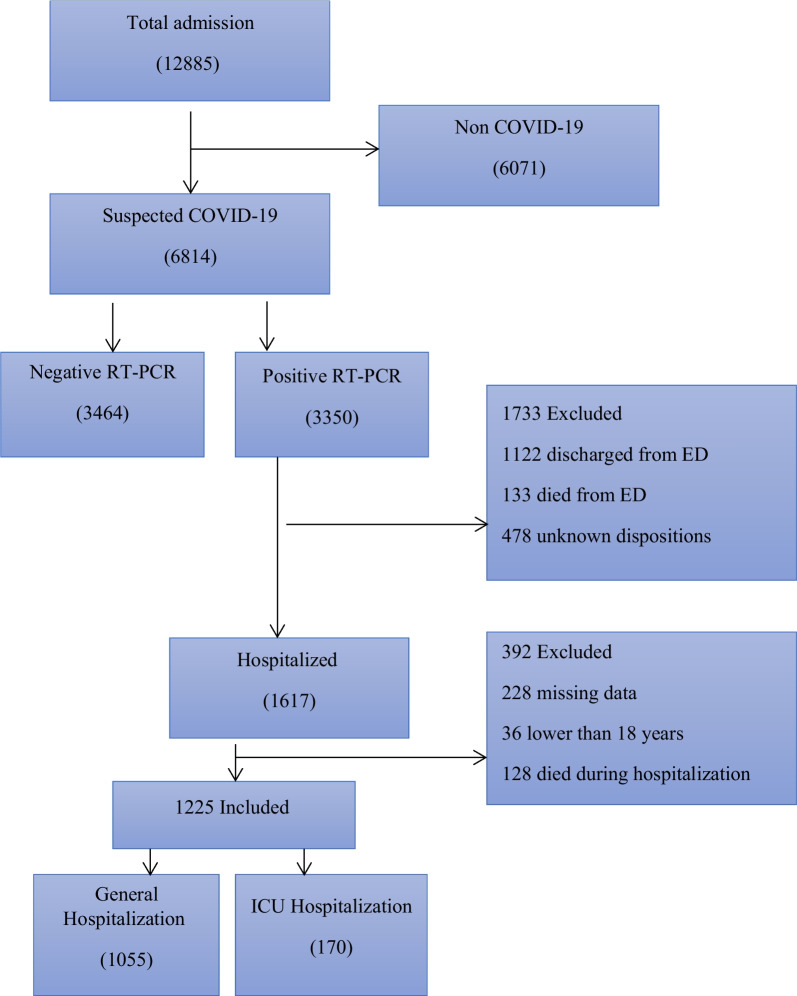
Flow chart describing patient selection

To overcome the impact of missing data on the models' predictive performance, all records containing missing data (more than 70%) were excluded from the analysis (n = 228). In addition, the remaining missing values were imputed with the mean or mode of each variable. Noisy and abnormal values, errors, duplicates, and meaningless data were assessed by researchers in collaboration with two infectious diseases specialists. For different interpretations of data preprocessing, we contacted the corresponding physicians. After applying the inclusion/exclusion criteria, 1225 records were entered into the study (Fig. 1). A significance level of p < 0.02 was used throughout the study.

### Predictor and outcome variables

In the database, a total of 53 variables were obtained for each patient, including demographics (five variables), clinical manifestations (14 variables), comorbidities/risk factors (seven variables), laboratory results (26 variables), and treatment intervention (one variable) (Table 1).

**Table 1:** Baseline predictor and outcome variables

Data classes	Predictors
Demographics	Gender, age, weight, height, and blood type
Clinical manifestations	Cough, contusion, nausea, vomiting, headache, gastrointestinal (GI) symptoms, muscular pain, chill, fever, dyspnea, loss of taste, loss of smell, runny nose, and sore throat
Comorbidities diseases/risk factors	Pneumonia, cardiovascular disease (CVD), hypertension, diabetes, cigarette smoking, alcohol consumption, and other underlying diseases
Laboratory results	Creatinine, red blood cell (RBCs) count, white blood cell (WBCs) count, hematocrit, hemoglobin, platelet count (PLT), absolute lymphocyte count (ALC), absolute neutrophil count(ANC), calcium, phosphorus, magnesium, sodium (Na +), potassium (K +), blood urea nitrogen(BUN), total bilirubin, aspartate aminotransferase(ASP), alanine aminotransferase(ALT), albumin, glucose, lactate dehydrogenase(LDH), activated partial thromboplastin time (PTT), prothrombin time (PT), alkaline phosphatase (ALP), erythrocyte sedimentation rate(ESR), C-reactive protein (CRP), hypersensitive troponin
Treatment intervention	Oxygen therapy
Outcome variable	Hospital LOS (days)

Hospital LOS is considered the outcome variable. This measurement of duration is a continuous variable calculated using the number of days from the time patients were admitted to the hospital until they were either discharged, referred to another hospital, or died while still in the hospital.

### Feature selection

Feature selection or variable selection is an effective technique that is used to determine the most meaningful variables, reduce the dimensions of the dataset, and improve the efficiency of ML algorithms [46]. In this study, variables with a correlation coefficient value less than 0.2 (P-value < 0.2) were identified as effective risk factors in predicting the LOS of COVID-19 patients and were included in the ANN models.

### Model development

An ANN is a set of computing algorithms that emulate the functions of biological neural networks. The components of the models are nodes, weights, and layers (input, hidden, and output layers) (41). MLP-ANN is the simplest and most commonly used ANN architecture due to its structural flexibility, good representational capabilities, and a large number of training algorithms [47, 48]. In this study, in order to develop an MLP-ANN, we used 12 training algorithms, including Levenberg–Marquardt (LM), Bayesian regularization (BR), Broyden-Fletcher-Goldfarb-Shanno (BFGS) Quasi-Newton, resilient backpropagation (RP), scaled conjugate gradient (SGC), conjugate gradient with Powell/ Beale (CGB) restarts, conjugate gradient Fletcher-Powell (CGF), conjugate gradient with Polak-Ribiére (CGP) updates, one step secant (OSS), gradient descent variable learning rate (GDX), gradient descent with momentum (GDM), and gradient descent (GD) backpropagation described in Table 2. Moreover, selecting the optimal number of neurons in the hidden layer is an important and difficult issue due to its effect on the performance and efficiency of ANNs. Therefore, the optimal number of neurons in the hidden layer is determined by constructing ANNs with a different number of neurons in the hidden layer. In this study, it was attempted to consider the basic parameters of the algorithms as the same so that the effect of choosing the learning algorithm on the performance of the network could be thoroughly investigated. All simulations were implemented using the full-featured dataset, including 53 features, and a derived dataset with 20 features after performing feature selection.

**Table 2:** The parameters of the selected ANN training algorithms

Training algorithm	MatLab function	Maximum number of epochs to train	Other parameters
LM	Trainlm	1000	Mu: 0.001
BR	Trainbr	1000	Mu: 0.005
BFGS	Trainbfg	1000	Name of the line search routine to use = srchcha Initial step size = 0.01
OSS	Trainoss	1000	Name of the line search routine to use = srchcha Initial step size = 0.01
SCG	Trainscg	1000	
CGB	Traincgb	1000	Name of the line search routine to use = srchcha Initial step size = 0.01
CGF	Traincgf	1000	Name of the line search routine to use = srchcha Initial step size = 0.01
CGP	Traincgp	1000	Name of the line search routine to use = srchcha Initial step size = 0.01
GDX	Traingdx	1000	Learning rate = 0.01 Momentum constant = 0.9
GDM	Traingdm	1000	Learning rate = 0.01 Momentum constant = 0.9
GD	Traingd	1000	Learning rate = 0.01
RP	Trainrp	1000	Learning rate = 0.01

### Model evaluation

The performance of each model was evaluated based on the RMSE for predicting LOS using a tenfold cross-validation method. This method trains and evaluates ML algorithms by dividing the dataset into a training partition used to train the models and a test partition used to validate the models' performance [49, 50]. In our study, to circumvent probable bias in the presentation order of the sample patterns to the ANN, the dataset was randomly divided into 90% for training and 10% for testing.

After identifying the best neural network training algorithm and the optimal number of neurons in the hidden layer for LOS prediction, in order to perform external validation, we conducted a three-month prospective study in Ayatollah Taleghani Hospital. The best model was applied to predict the LOS of all hospitalized patients confirmed to have COVID-19 via PCR testing and admitted to this hospital from February 1, 2021 to April 30, 2021 (343 patients). The comparison between the output of the selected neural network and the real data as a benchmark was conducted by calculating the RMSE.

### Ethical considerations

This study was approved by the Ethical Committee Board, Abadan University of Medical Sciences (code: IR.ABADANUMS.REC.1399.222). To protect patients’ privacy and confidentiality, we concealed the unique identifying information of all patients during the process of data collection and presentation.

## Results

### Characteristics of COVID-19 patients

In this study, a retrospective analysis was conducted on the medical records of 1225 COVID-19-positive patients evaluated between January 9, 2020 and January 20, 2021 at Ayatollah Taleghani Hospital, and it was revealed that 664 (54.20%) patients were male and 561 (45.80%) were female. The overall mean age was 57.25 (interquartile 18–100) years. A total of 170 (13.87%) patients were hospitalized in the ICU and 1055 (86.13%) were hospitalized in general wards. Descriptive statistics for the 1225 records in this dataset are shown in Table 3.

**Table 3:** Summary of COVID-19 patients' characteristics

Qualitative variables	Values	Frequencies
Blood Type	A−	17
	A +	552
	B−	13
	B +	126
	O−	29
	O +	421
	AB−	6
	AB +	61
Gender	Male, Female	664, 561
Cough	Yes, No	958, 267
Contusion	Yes, No	409, 816
Nausea	Yes, No	401, 824
Vomiting	Yes, No	346, 879
Headache	Yes, No	312, 913
GI symptoms	Yes, No	252, 973
Muscular pain	Yes, No	623, 602
Chill	Yes, No	591, 634
Fever	Yes, No	628, 597
Pneumonia	Yes, No	1044, 181
Oxygen therapy	Yes, No	1053, 172
Dyspnea	Yes, No	1078, 147
Loss of taste	Yes, No	272, 953
Loss of smell	Yes, No	305, 920
Runny Nose	Yes, No	437, 788
Sore throat	Yes, No	444, 781
Other underlying diseases	Yes, No	735, 490
CVD	Yes, No	306, 919
Hypertension	Yes, No	395, 830
Diabetes	Yes, No	268, 957
Cigarette smoking	Yes, No	41, 1184
Alcohol consumption	Yes, No	11, 1214
CRP	Positive, Negative	1063, 162
Hypersensitive troponin	Positive, Negative	58, 1167
Quantitative variables	Range	Mean (SD)
Age (year)	18–100	57.25 (17.8)
Height	92–195	168.53 (8.5)
Weight	6.5–163	75.20 (13.0)
Creatinine	0.1–17.9	1.39 (1.4)
RBC	1.38–13.1	4.56 (0.9)
WBC	1300–63,000	8182.34 (4897.4)
Hematocrit	3.6–73.9	39.20 (6.7)
Hemoglobin	3.7–46	13.21 (2.4)
Platelet count	108,000–691,000	215,493.66 (88,380.1)
ALC	2–95	23.74 (11.8)
ANC	8–98	74.52 (12.3)
Calcium	0.9–14.1	9.68 (0.8)
Phosphorus	2–12.4	3.50 (0.5)
Magnesium	1.14–19.1	2.16 (0.6)
Sodium	37–157	137.94 (5.3)
Potassium	2.5–14.2	3.98 (0.7)
BUN	0.5–251	42.52 (31.7)
Total bilirubin	0.01–10	0.72 (0.7)
AST	3.8–924	44.45 (53.5)
ALT	2–672	38.29 (41.6)
Albumin	0.2–8.9	4.02 (0.5)
Glucose	18–994	136.09 (74.2)
LDH	4.6–6973	555.68 (339.0)
Activated PTT	1–120	28.56 (11.4)
PT	0.9–46.8	12.82 (1.9)
ALP	9.6–2846	213.12 (139.2)
ESR	2–258	40.65 (28.8)

### Variables included in the ANN models

The results of feature selection for determining the most important diagnostic criteria affecting COVID-19 hospital LOS based on the correlation coefficient at P < 0.2 are demonstrated in Table 4.

**Table 4:** The selected features affecting COVID-19 hospital LOS

Variables	Pearson correlation	P-value
Age	− 0.045	0.119
Creatinine	− 0.066	0.019
WBC	− 0.054	0.057
ALC	− 0.057	0.044
ANC	0.061	0.033
Calcium	− 0.055	0.055
BUN	− 0.059	0.038
ASP	0.054	0.057
ALT	0.047	0.097
Cough	0.299	0.041417
Hypertension	− 0.1744378	0.0541
CVD	0.2746594	0.125
Diabetes	0.0980716	0.104
Dyspnea	0.4443414	0.017
Oxygen therapy	0.460136	0.008
Pneumonia	0.2690936	0.115
LDH	0.056	0.049
GI complications	− 0.20181	0.179
ESR	0.040	0.157
CRP	0.0258788	0.196

After feature selection, a total of 20 features acquired the determined correlation coefficient at P < 0.2. Features including age, creatinine, white blood cell (WBC) count, lymphocyte /neutrophil count, blood urea nitrogen (BUN), aspartate aminotransferase (ASP), alanine aminotransferase (ALT), lactate dehydrogenase (LDH), activated partial thromboplastin time (PTT), coughing, hypertension, cardiovascular disease (CVD), diabetes, dyspnea, oxygen therapy, pneumonia, gastrointestinal (GI) complications, erythrocyte sedimentation rate (ESR), and C-reactive protein (CRP) were identified as the most significant factors for predicting hospital LOS.

### Determining the appropriate configuration for the MLP

To determine the best predictive model, different MLP networks with multiple configurations were trained and their performance was evaluated using tenfold cross-validation. Tables 5 and 6 list the RMSE rate of each network with different training algorithms and the number of neurons in the hidden layers for both datasets.

**Table 5:** The RMSE of training algorithms for the full-featured dataset

Training algorithm	The number of neurons					
	5	10	15	20	25	30
LM	2.3338	2.3825	2.4444	2.3863	2.3782	3.0873
BR	1.9291	1.732	1.6963	1.6213	1.9454	1.8312
BFGS Quasi-Newton	2.3948	2.3701	2.3726	2.3932	2.2855	2.2507
RP	2.3513	2.3365	2.4433	2.6511	3.2506	2.8938
SCG	2.4085	2.4049	2.3882	2.3791	2.3944	2.322
CGB	2.3927	2.4045	2.4045	2.4301	2.3861	2.3365
CGF	2.4015	2.3845	2.3936	2.3581	2.3642	2.319
CGP	2.3966	2.3989	2.4514	2.4038	2.3545	2.7662
OSS	2.3706	2.3924	2.4454	2.4101	2.5861	2.8005
GDX	2.405	2.3985	2.8746	2.8096	2.6103	2.9711
GDM	16.0903	23.6745	26.0844	25.2706	32.1784	41.2996
GD	16.896	42.1661	36.6554	30.9327	26.1043	29.3946

**Table 6:** The RMSE of training algorithms for the dataset with selected features

Training algorithm	The number of neurons					
	5	10	15	20	25	30
LM	2.3617	2.4407	2.3937	2.3346	2.3564	2.4074
BR	2.3386	2.2332	2.3493	2.2995	2.2965	2.2962
BFGS Quasi-Newton	2.3756	2.3388	2.3847	2.3284	2.8562	2.757
RP	2.3998	2.3869	2.4774	2.7853	3.296	2.7826
SGC	2.5182	2.4187	2.3952	2.9402	2.3695	2.4256
CGB	2.3895	2.372	2.3759	2.3617	2.3459	2.3962
CGF	2.3851	2.4322	2.4218	2.4023	2.3655	2.4033
CGF	2.4646	2.4124	2.4607	2.3935	2.4053	2.4818
OSS	2.3997	2.3854	2.4639	2.4297	2.5942	2.353
CHF	2.4135	2.4264	2.5287	2.9861	3.3325	3.6619
GDM	13.7549	17.353	46.4296	33.9491	27.4377	54.9474
GD	9.1643	18.8733	50.1962	53.7368	25.1357	61.4867

**Fig. 2:**
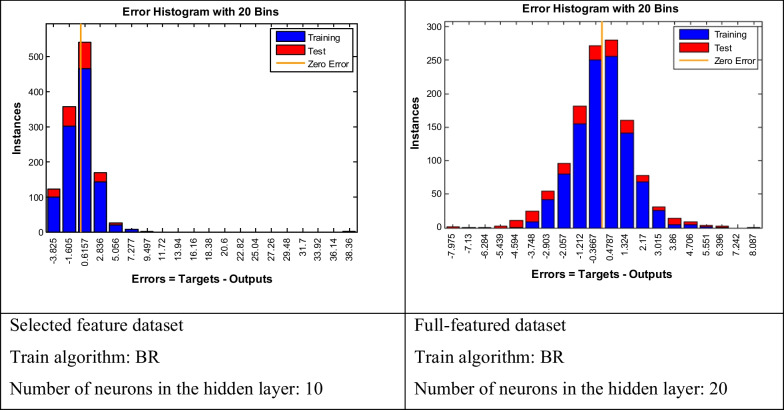
Error histogram for the best model

According to Tables 5 and 6, using a total of 53 risk factors, the best results were obtained by the neural network with 20 neurons in the hidden layer and the BR training algorithm. The RMSE of this technique was 1.6213, which was the lowest error rate among the designed networks. The results also showed that based on the selected features (n = 20), the neural network with the BR training algorithm and 10 hidden neurons achieved the best result (RMSE = 2.2332). The error histograms for these two models are depicted in Fig. 2.

**Fig. 3:**
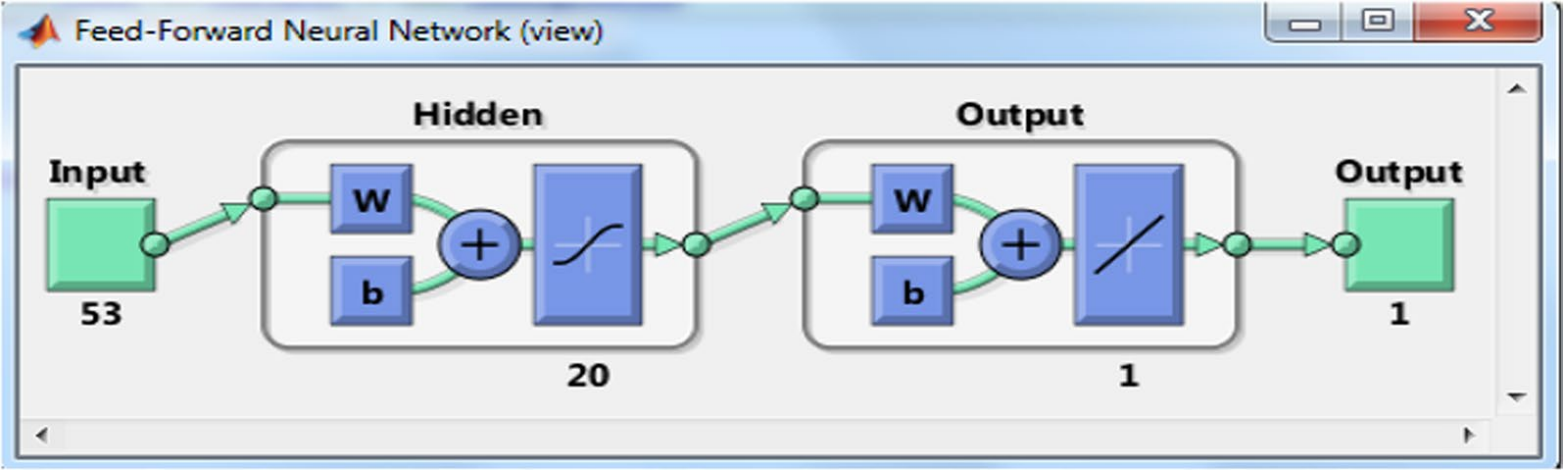
The architecture of the BR training algorithm with 20 neurons used for COVID-19 LOS prediction

According to Fig. 2, although the error rate in the dataset with selected features is higher, the error distribution is better, and for small samples, it indicates an error greater than the CDC threshold (i.e., three days). The network architecture for the BR training algorithm based on the whole dataset is shown in Fig. 3.

**Fig. 4:**
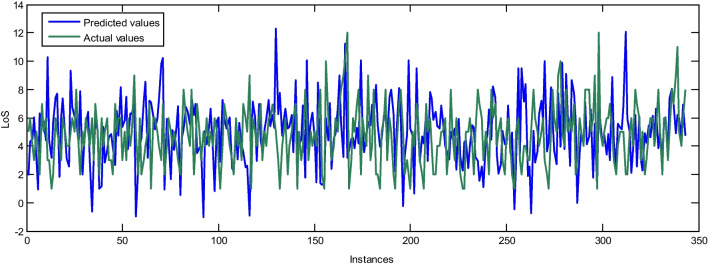
Comparison between the output of the best neural network and the actual data for the external validation sample

For external evaluation, the best LOS prediction model was utilized for the prospective study and predicted the LOS of patients with an RMSE of 2.8529. Figure 4 compares the actual and predicted values of LOS for the external validation cases using the MLP with BR as a training function and 20 neurons in the hidden layer.

**Fig. 5:**
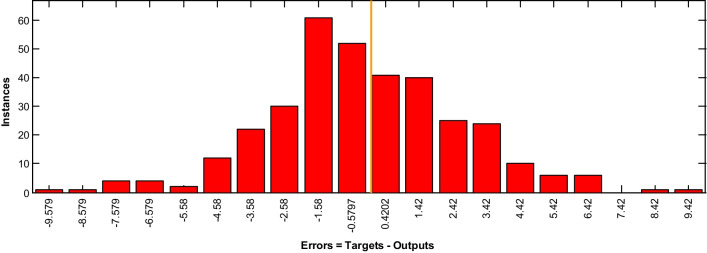
Error histogram of the LOS prediction model for the external validation sample

The error histogram of the external validation (Fig. 5) showed that the proposed model has a good ability to predict LOS of hospitalized COVID-19 patients, and for small samples, it indicates an error of more than two days.

## Discussion

In this study, we developed and evaluated several MLP neural networks training algorithms to predict the LOS of COVID-19 patients using full and selected feature datasets (53 and 20 features, respectively). The experimental results revealed that BR had the best performance compared to the other techniques in LOS prediction for COVID-19 patients with an RSME of 1.6213 (layer 20) and 2.2332 (layer 10) for the whole and selected feature datasets, respectively. In the present study, the most important variables (n = 20 predictors) were identified through a correlation coefficient at the level of P-value < 0.2. These variables include age, creatinine, WBC, lymphocyte/neutrophil count, BUN, ASP, ALT, LDH, activated PTT, coughing, hypertension, CVD, diabetes, dyspnea, oxygen therapy, pneumonia, GI complications, ESR, and CRP.

Determining the best network training algorithm depends on many factors, including the complexity of the problems, the amount of data in the training set, the number of weights and biases in the network, the error goal, and whether the network is used for pattern recognition or function approximation [51, 52]. In our study, the LM training algorithm also exhibited a satisfactory performance in estimating the functions. If LM is trained with low number weights, it will converge more quickly and have a much lower error rate than other training algorithms. But when it is trained with a high number weight, its efficiency will decrease [53, 54]. Conjugate gradient-based training algorithms (SCG, CGB, CGF, and CGP) identical to LM have a good performance in estimating the functions and recognizing patterns. Furthermore, their efficiency would not reduce significantly with an increase in the weight number [55, 56]. In the present study, algorithms based on LM as well as SCG, CGB, CGF, and CGP had a satisfactory performance. The RP algorithm had undesirable performance in function approximation compared to other training algorithms because it demonstrated better capability for pattern recognition [57, 58]. The BFGS Quasi-Newton does not require as much storage as LM, but the computations required increase geometrically with the size of the ANN because the inverse matrix must be calculated for each iteration. The GDX had a slow convergence and the other two gradient descent algorithms (GDM and GD), as shown in this study, do not perform well in function approximation [59, 60]. The BR training algorithm updates network weights and biases using the LM optimization method. It minimizes the combination of squares error and weights and seeks the right combination that leads to a network with high generalizability [61, 62]. Since BR looks for a network with high generalizability, in our study, the best results were obtained by this training algorithm.

Similarly, Conde-Gutie´rrez et al. used the ANN method in their study to model and predict the cumulative number of deaths from COVID-19 in Mexico. They applied LM, BFGS, and batch GD training algorithms to fit coefficients (weights and biases). The comparison between the real data and those attained by the ANN model when using the training algorithms indicates satisfactory correlations with RMSEs of 0.2290, 0.2165, and 0.7722, respectively. Based on the computation time, the LM algorithm is the most appropriate for modeling the dynamics of deaths from COVID-19. The LM algorithm estimated the coefficients in the shortest probable time (46.23 s) while the BFGS Quasi-Newton algorithm showed better precision fits for the real data. The batch GD algorithm had the least capacity to model the real data and needed more neurons in the hidden layer [63]. Namasudra et al. presented a nonlinear autoregressive (NAR) neural network time series (NAR-NNTS) model for predicting COVID-19 cases. This NAR-NNTS model is trained with SCG, LM, and BR training algorithms. The results showed that the NAR-NNTS model trained with LM performs better than other models for COVID-19 epidemiological data prediction [64]. Sapon et al. used the data of 250 diabetic patients to train the network to identify the disease pattern. They used three training algorithms including BR, BFGS, and LM. The BR algorithm had the best performance in the prediction of diabetes compared to the BFGS Quasi-Newton and LM algorithms. The BFGS Quasi-Newton algorithm possessed 0.86714 correlation coefficients with 578 epochs while the BR algorithm acquired 0.99579 for 37 epochs and LM held 0.6051 for only five epochs. Therefore, according to their study, the BR algorithm presented a good correlation between the estimated targets and actual outputs (i.e., 0.99579) with 88.8% prediction accuracy, confirming the validation that shows the correctness of this algorithm to perform effective diabetes prediction [65]. Narayan et al. compared three training algorithms, including, LM, RP, and GDM, for training the network to estimate clinical gait mechanics. The results of correlation coefficients revealed the significant potential of the LM model over RP and GDM models while estimating the gait mechanics [66]. Using the data of 303 samples to predict heart diseases, Karim et al. compared different training algorithms, including GD, GDM, RP, SCG, CGP, CGF, BFGS Quasi-Newton, and LM. According to their findings, BFGS Quasi-Newton training algorithm is the most suitable for the development of an ANN prediction model for heart diseases because of its optimal speed and accuracy [67].

Many studies have shown that certain characteristics are associated with hospital LOS [8, 13, 14, 18, 19, 68, 69]. The most important clinical variables affecting longer LOS in reviewed studies include age (older age) [13, 18, 19, 69], comorbidities [8, 14, 68, 69] (CVD, hypertension, diabetes, and respiratory diseases, such asthma or chronic obstructive pulmonary disease (COPD)), loss of consciousness [8, 14, 69], increased BUN [8, 14, 18, 19], leukocytosis [8, 68], decreased oxygen saturation (SPO_2_) [13, 18, 19, 68], mechanical ventilation (oxygen therapy) [8, 14, 69], pleural effusion [13, 19, 68], dry cough [13, 69], and fever [8, 18, 19, 69]. In general, high compliance was observed from the results of classifying and prioritizing variables in reviewed studies with the most common variables selected in the current study. The results of our study indicated that the designed ANN model can effectively predict the COVID-19 patients’ LOS by using clinical variables that are readily available at the first time of admission.

## Limitations

Despite the strength of the algorithms presented, the novelty of the approach, and the promising predictive results, the study had some limitations that should be recognized. First, we dealt with a retrospective dataset that might suffer from uneven or imbalanced, noisy, duplicate, and meaningless values, which may skew results. Thus, the dataset was balanced by eliminating confounding factors as much as possible. Second, this study was performed at a single regional center and was only based on 1225 records; therefore, the results may not be generalizable and may confine the model’s applicability to other contexts. Although we only used the ANN algorithm for prediction analyses, other algorithms may perform better. As the analyses were based on a particular cohort of COVID-19 (alpha variant) before the building of vaccines, this limits the applicability for modern usage, especially in terms of delta and omicron variants and a vaccinated population may be limited. Lastly, the model developed in our study is limited to features commonly available at the initial time of admission. Although this is consistent with the aim of our study to predict COVID-19 LOS based on the admission data, the features generated during the hospitalization, such as radiological, imaging, and therapeutic intervention features, may improve the results of the models. The performance of our computational model can be improved in the future, if we examine more ML techniques at prospective, multicenter, and qualitative datasets.

## Conclusions

Predicting LOS allows hospitals to assess the overall patient load, which in turn allows improved scheduling of patient admissions, leading to a reduced variation of bed occupancies in hospitals. Estimating the LOS of hospitalized patients with COVID-19 is crucial for effectively planning bed management along with related personnel and facilities requirements. The results showed that MLP with the BR training algorithm has a better performance than the other models. With further validation, our models are expected to serve as objective, measurable, and evidence-based tools to predict COVID-19 LOS and optimize the use of limited hospital resources. While our models are trained using a dataset from one hospital, they can be retrained using a multicentral dataset from different geographic locations, which would improve the generalizability of the models to predict COVID-19 patients’ LOS. For future studies, it is suggested to train the ANN models with multicentral datasets. This would assist in improving the learning capability of the models as the trained dataset will be more diversified, hence providing better predictive performance for the models.

## Data Availability

All data generated and analyzed during the current study are not publicly available but are available from the corresponding author on reasonable request and the Student Research Committee of Abadan University of Medical Sciences approval.
